# Kinematic coordinations capture learning during human–exoskeleton interaction

**DOI:** 10.1038/s41598-023-35231-3

**Published:** 2023-06-26

**Authors:** Keya Ghonasgi, Reuth Mirsky, Nisha Bhargava, Adrian M. Haith, Peter Stone, Ashish D. Deshpande

**Affiliations:** 1 Department of Mechanical Engineering, The University of Texas at Austin, Austin, TX USA; 2 Department of Computer Science, Bar-Ilan University, Ramat Gan, Israel; 3 Department of Mechanical Engineering, Carnegie Mellon University, Pittsburgh, PA USA; 4 Department of Neurology, Johns Hopkins University, Baltimore, MD USA; 5 Department of Computer Science, The University of Texas at Austin, Austin, TX USA; 6 Sony AI, Austin, TX USA

**Keywords:** Mechanical engineering, Musculoskeletal system, Scientific data

## Abstract

Human–exoskeleton interactions have the potential to bring about changes in human behavior for physical rehabilitation or skill augmentation. Despite significant advances in the design and control of these robots, their application to human training remains limited. The key obstacles to the design of such training paradigms are the prediction of human–exoskeleton interaction effects and the selection of interaction control to affect human behavior. In this article, we present a method to elucidate behavioral changes in the human–exoskeleton system and identify expert behaviors correlated with a task goal. Specifically, we observe the joint coordinations of the robot, also referred to as kinematic coordination behaviors, that emerge from human–exoskeleton interaction during learning. We demonstrate the use of kinematic coordination behaviors with two task domains through a set of three human-subject studies. We find that participants (1) learn novel tasks within the exoskeleton environment, (2) demonstrate similarity of coordination during successful movements within participants, (3) learn to leverage these coordination behaviors to maximize success within participants, and (4) tend to converge to similar coordinations for a given task strategy across participants. At a high level, we identify task-specific joint coordinations that are used by different experts for a given task goal. These coordinations can be quantified by observing experts and the similarity to these coordinations can act as a measure of learning over the course of training for novices. The observed expert coordinations may further be used in the design of adaptive robot interactions aimed at teaching a participant the expert behaviors.

## Introduction

Robotic exoskeleton-based training has the potential to affect persistent changes in human behavior^1^ making these devices uniquely suited for rehabilitation post neurological injury^2–4^. For example, Harmony^5^ is a bi-manual upper-body exoskeleton developed for assessment^6,7^ and rehabilitation^8,9^ post neurological injuries such as stroke. Robots like the Harmony exoskeleton allow for reliable sensing and robust control of close interactions between the human body and the robotic system. Though there has been significant progress in designing robots for rehabilitation, applying these robots to train human behavior remains an open challenge due to the difficulty of predicting the effect of physical interaction on the combined human–robot system’s behavior^10^. The complexity of the human neuromuscular system and its stochastic motor behaviors coupled with the dynamics of the exoskeleton limits our understanding of this interaction. In this article, we address the challenge of quantifying human–exoskeleton interaction through the observation of kinematic behavioral changes compared to a known successful coordinated behavior. We demonstrate the efficacy of our method in the context of novel motor task training. We further explore the use of our metric to quantify domain knowledge in the form of expert behaviors learned for a given task goal. Characterizing human–robot interaction effects using our method could significantly improve the assessment of robotic rehabilitation and translate expert performance to effective robot interactions for human learning, where the robot can encourage the patient to move in a manner closer to the known successful coordinations^11,12^.

**Figure 1:**
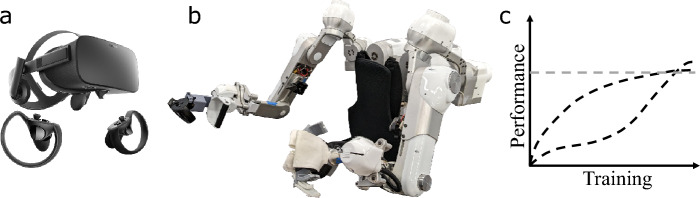
We observe the effect of learning in (**a**) the task space (score and end effector speed) measured in a virtual task environment, and (**b**) the joint space (joint angles, velocities, and torques) measured using the Harmony exoskeleton’s sensors. The expected trends in performance change over the course of training seen in (**c**) may vary, but participants are all expected to improve and become experts crossing a threshold performance level.

The field of motor learning approaches the challenge of quantifying the neuromuscular system’s behavior through observation of performance. Research in this area largely focuses on cognitive learning^13,14^ or relatively simple static motor learning^15–17^. However, dynamic behaviors that are representative of day-to-day motor learning and neurorehabilitation have received less attention^18,19^, and even fewer studies consider dynamic interactions between a human and an exoskeleton^20–22^. Dynamic task studies use “extrinsic” performance metrics such as task success or accuracy^23–25^, which are easy to measure and accurately represent the effect of human movement on task outcome. But these metrics fail to capture the underlying neuromuscular behavior that affects the performance. “Intrinsic” metrics instead capture the underlying motor behavior for example by considering joint kinematics^26,27^. Thus, intrinsic metrics together with extrinsic metrics afford a more complete picture of performance by observing both human behavior and task outcomes respectively. In this article, we perform this comprehensive analysis using extrinsic metrics from the task environment (such as virtual reality or camera-based hand-tracking tasks, Fig. 1a) and intrinsic metrics from the Harmony exoskeleton (Fig. 1b). We aim to develop generalizable metrics of performance that improve as training progresses (Fig. 1c), thereby allowing us to characterize learning.

The study of intrinsic metrics has shown that humans employ dimensionality reduction to simplify neuromuscular control. For example, humans coordinate their joints to move in a smooth and controlled manner despite over-actuation in the joint space for cartesian-space tasks like reaching^28–30^. These coordination behaviors, also referred to as kinematic coordinations, are time-independent features of behavior exhibited during a movement. Principal component analysis (PCA) is commonly used to identify these coordinations in motor learning literature. The same method has been used in the control of exoskeletons to imitate a physical therapist’s assistance^31^, to discourage certain “unhealthy” coordination behaviors^22^, or for assistive path planning^32^. These studies have shown kinematic coordination behaviors may emerge through, and can be affected by, human–exoskeleton interaction. However, these studies don’t consider the behavioral information encoded within these coordinations. We posit that these joint coordinations emerge in exoskeleton interactions as a consequence of learning and may provide a potential avenue for exoskeleton intervention design based on expert behaviors. The novel idea of this article is to elucidate task-specific domain knowledge in the form of expert interaction behaviors through kinematic coordination behaviors. Specifically, we present a method to quantify the human–robot system’s joint coordinations, identify expert task-specific behaviors, and measure similarity to these desired coordinations over the course of learning.

In this article, we show that humans learn certain synergistic behaviors over time and that task goals, task instructions, and targeted practice all affect the learned behavior. We validate kinematic coordination behaviors as a measure of the human–robot system’s dynamic interaction behavior through four results across three human-subject studies using the Harmony exoskeleton. First, we show that participants learn a novel dynamic task to an equivalent extent both with and without the exoskeleton as measured by changes in extrinsic metrics *within participants* (Result 1). This result indicates that the robot does not deter learning, further verifying it as a suitable platform for the study of human–robot interaction through novel task learning. Next, we show that successful behaviors employed by *a given participant* tend to converge as learning occurs (Result 2). Participants appear to learn joint coordinations that are correlated with success and learn to employ them more effectively. Third, we compare these learned “successful” coordinations to other movement attempts of the same task and find convergence to these *participant-specific* coordination behaviors through the course of training regardless of whether participants achieve success (Result 3). Finally, we construct task-specific kinematic coordination behaviors from learned successful attempts *across different participants* and find that experts converge to these coordinations for certain task goals (Result 4). These constructed coordinations present a novel method to quantify domain knowledge toward robot intervention design. For each of the results 2, 3, and 4, a reference coordination behavior is defined, either for a given participant or task goal, and compared to other movement attempts using a kinematic coordination distance metric such that a lower distance indicates a similarity to the reference joint coordination behavior. Together, these results show that kinematic coordinations are good indicators of learning, and further, that these coordinations encode expert behaviors that may be beneficial to the design of robotic interventions for exoskeleton-based motor training.

**Figure 2:**
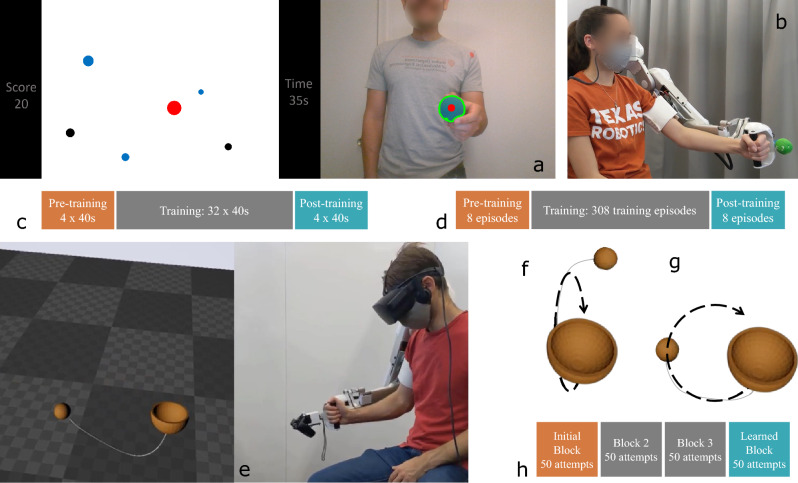
Reach ninja task (**a**,**b**) a webcam tracks the movement of an object held in the player’s hand. The player’s goal is to maximize their score by moving the red cursor (tracking their hand movement) to hit the blue (positive) targets while avoiding the black (negative) targets. Participants train for roughly the same amount of time on this task in study 1 without the exoskeleton (**a**,**c**) and in study 2 while wearing the exoskeleton (**b**,**d**). Virtual simplified Kendama task (**e**) on the left is the task as observed through the VR headset while on the right a participant attempts the task in VR while wearing the Harmony exoskeleton. The goal of the task is to move the Kendama cup so that the ball is swung up and caught in the cup. (**f**) and (**g**) show the front-swing and side-swing strategies respectively. Participants are trained on at least 200 attempts in 4 blocks of 50 each (**h**).

In the following sections, we present a comprehensive analysis of kinematic coordination as an intrinsic performance metric for the characterization of human–exoskeleton interaction behaviors. We find that humans *learn* to coordinate the joints of the Harmony exoskeleton to leverage its dynamics. Further, the manner of coordination converges as task performance improves, suggesting that these observed coordination behaviors are a measure of the implicitly learned behavior. Finally, we identify task-specific coordinations that some expert participants converge to over time. Our overarching goal is to use the information encoded in these kinematic coordinations to motivate exoskeleton interventions for motor training and rehabilitation. The same robot that measures the kinematic coordinations may *teach* expert behaviors to novices, through carefully designed interaction control, potentially resulting in faster and larger improvements in task performance. The characterization of dynamic task learning using kinematic coordination behaviors opens up many novel avenues for robot-based training by targeting the learning of coordinations rather than extrinsic performance improvement, including coordination-based training effect assessment or coordination-focused robot intervention design. Such coordination-focused approaches may be applied to exoskeleton-based rehabilitation^12^ as well as the design and control of other physical human–robot interaction devices like robotic prostheses and surgical robots.

## Results

To facilitate our study of human behavior through motor training, we selected tasks that were both dynamic and challenging, ensuring learning through repeated practice. Two task platforms are used in our studies: (1) the 2-D video game Reach Ninja^33^ (Fig. 2a,b), and (2) a simplified virtual reality implementation of the Kendama task^34^ (Fig. 2e). In the Reach Ninja task, the individual’s goal is to maximize their score by using the red hand-tracking cursor to hit the positive scoring (blue) targets while avoiding the negative scoring (black) ones. In the virtual kendama task, participants aim to swing the ball attached to a cup through a string and catch it in the cup. Participants are trained to perform the task using two distinct strategies: front swing (Fig. 2f) and side-swing (Fig. 2g). Both of these tasks require the participants to learn a new dynamic motor behavior, rather than adapt from previously learned tasks. These tasks are complementary to one another on at least three dimensions: (a) the Reach Ninja task is sequential and requires the human to learn a long-term strategy, as opposed to the short-term dynamic Virtual Kendama task; (b) in Reach Ninja the end effector is in the human’s hand, while in Virtual Kendama the controller is a rigid extension of the robot end effector; and (c) in Reach Ninja the human needs to reason about several objects that move in the environment, whereas in Kendama there is only one moving object. These differences mean that together, the two tasks cover a wide variety of potential properties that can affect learning. Thus, by observing behavioral changes due to learning in these two domains, we validate the robustness of task-dependent extrinsic metrics and generalizable intrinsic metrics to identify behavior changes due to learning regardless of these varying properties. In particular, we demonstrate the generalizability of our kinematic coordination metric to both task domains despite the differences in the task goals and movement behaviors.

We present results from three human-subject studies. The first study considers novel task learning without robot interaction, while the other two consider learning while using the Harmony exoskeleton^5^. In both robot studies presented in this article, the Harmony exoskeleton is set to a gravity-assistance mode where it passively follows the wearer’s movements while accounting for its own weight. Only naive participants unfamiliar with the tasks are recruited for the experiments. Extrinsic performance metrics are used to evaluate performance changes before and after the training to confirm learning and intrinsic metrics are designed to characterize behavioral changes as learning occurs. The three studies presented below in detail are Reach Ninja, no robot (Study 1: RNNR, Fig. 2c), Reach Ninja, with robot (Study 2: RNWR, Fig. 2d), and Virtual Kendama, with robot (Study 3: VKWR, Fig. 2h). We use repeated measures ANOVA for statistical analysis and reject the null hypothesis on the significance condition $p < \alpha$ where $\alpha = 0.05$. All statistically significant results are highlighted in Table 1. Note that not all hypotheses presented in the Methodology section are discussed in the manuscript. The hypotheses that are not discussed did not show a significant result, thereby not allowing us to reject the null hypotheses. These results are omitted in the discussion as they do not add much and do not change the key contributions of the article. Further, we only present the p-values of the relevant hypotheses as they are sufficient to establish the validity of using kinematic coordination behaviors to capture learning effects. Future studies will also report effect size as we focus more on the learning effects themselves. The same details for the current results can be found in the supplementary material available along with the article.

### Result 1: Individuals learn to perform novel dynamic upper-body tasks while wearing an upper-body exoskeleton

The first milestone in this research is showing that donning an exoskeleton does not hinder learning when compared to a similar training process without the exoskeleton. We present results comparing two similar task-learning experiments with and without the Harmony exoskeleton. The goal of such a comparison is to establish that the exoskeleton is a feasible platform for the study of motor learning. Though the exoskeleton provides several sensing and actuation capabilities it does offer physical limitations in how dynamic tasks can be performed, such as with the available degrees of freedom and inertia effects. The comparison presented in this result shows that the exoskeleton has no direct detrimental effects on skill acquisition. The comparison of the two cases is presented with the Reach Ninja task. We also present improvements observed in training on the Virtual Kendama task while wearing the exoskeleton to establish the task is learnable.

**Figure 3:**
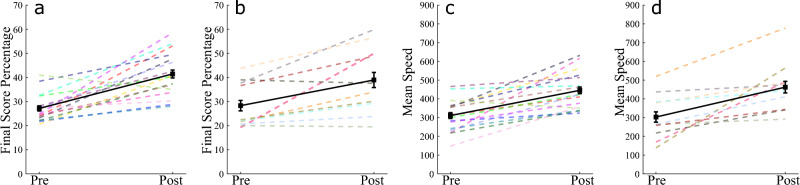
Extrinsic performance metrics on the Reach Ninja task. Each colored dashed line refers to a different participant. The solid black line with square markers shows the averages across participants in each block and the error bars show the standard error in the pre-training and post-training blocks respectively. The cross-effect of No Robot (**a**,**c**) versus With Robot (**b**,**d**) on pre- versus post-training performance shows a null result, suggesting that there was no significant effect of the exoskeleton on learning effects. The exoskeleton does not hamper task learnability.

#### Reach Ninja task

Participants learn to perform the Reach Ninja task under two different conditions: (1) without the robot (Fig. 2a), and (2) with the robot (Fig. 2b). In the no-robot case, participants practice a timed version of the task with their dominant arm, whereas in the robot case, participants practice an episodic version of the task with their non-dominant arm. The two experimental protocols are matched so that participants get roughly similar amounts of training time in-game (training episode length is approximately 4 s on average). Some results from each of these studies have been presented independently in prior works^33,35^. Here, we present a broader comparison between the results of the two studies to identify the effect of the exoskeleton on the changes in performance through repeated practice on the Reach Ninja task. Two extrinsic performance metrics are used to measure learning:

Final score percentage: final score of a given attempt as a percentage of the maximum possible score averaged across all attempts in a block (%).

Mean speed: average speed of the player’s hand during a given task attempt (px/s).

As presented in prior works^33,35^, in both studies, participants improve in the chosen extrinsic performance metrics (Fig. 3), demonstrating higher accuracy (final score percentage) and rigor or confidence (mean speed) in their movements with $p < \alpha$. Additionally, in this paper we present results from a two-way repeated measures ANOVA, with pre-training and post-training measurements as a within-subject factor and no-robot versus robot as a between-subject factor. Combining the results from these two studies shows no significant effect of the exoskeleton on learning ($p = 0.377 > \alpha$). This null result suggests that in both cases, with and without the exoskeleton robot, participants were able to learn the reach ninja task by themselves.

It should be noted that there were some differences in the overall experimental setup between the two cases in addition to the exoskeleton, particularly in the definition of an attempt, and the total experiment time. Further, in both cases, participants were given one of two types of training. However, as there was no effect of the training on the performance in either study, we consider all participants from one study to be part of a single group (either with or without the robot). With this larger number of participants, we still see no significant effect of the exoskeleton on the overall performance improvement. This null result does not necessarily confirm that the learning in the two experimental conditions is identical. However, as the training time was roughly the same, and the task goal and dynamics remained unchanged, we conclude that the robot had no significant negative effect on task learnability. This result validates our use of the exoskeleton to study learning and eventually as a platform for motor training for skill acquisition. We do not consider an in-depth analysis of the specific changes in the performance metrics here as it is beyond the scope of this paper. However, future analyses of the Reach Ninja task will include larger subject populations to increase our confidence in the statistical results. In the rest of the paper, we present results from human-subject experiments conducted with the exoskeleton.

**Figure 4:**
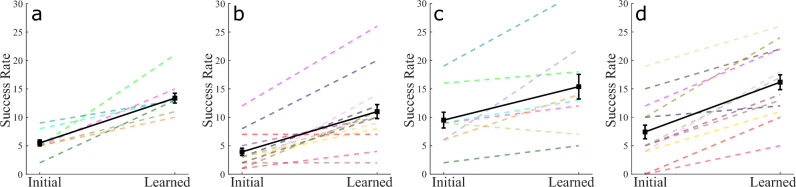
Success rate in the Virtual Kendama task: (**a**) Front swing in session A, (**b**) Side swing in session A, (**c**) Side swing in session B, (**d**) Front swing in session B. Results in (**a**) and (**c**) are from the same group of participants, and (**b**) and (**d**) are from the same group of participants. Each colored dashed line refers to a different participant. The solid black line with square markers shows the averages across participants in each block and the error bars show the standard error in the initial and learned blocks respectively. In all cases, session A or B, side swing or front swing strategy, participants improved their success rate (number of successes in a block of 50 attempts) from the initial (first 50) to the learned (last 50) attempts.

#### Kendama task

Participants train on the simplified virtual reality Kendama task by repeatedly attempting to perform the task while wearing the exoskeleton. A training session consists of 200 attempts split into four blocks of 50 with short breaks in between each block to prevent fatigue. Participants are asked to perform the task with one of two strategies, *side swing* or *front swing* during a session of the task. They then repeat the experiment on a different day using the other movement strategy. In total, data is collected for 21 participants performing two sessions of training with 200 attempts in each session, except for one participant for whom only data from the first session is considered. A total of 8 participants learn the task using the front swing strategy in their first session, and 13 participants learn the side swing strategy in their first session.

For each attempt, success is determined as a binary value: if the ball is caught in the cup and held for at least 1 s, the attempt is considered successful. To illustrate changes in the player’s performance over time, the number of successes in the first 50 attempts and the last 50 attempts is plotted in Fig. 4. The number of successes in the first 50 attempts is referred to as the *initial* success rate, while the number of successes in the last 50 attempts is referred to as the *learned* success rate. The change from the initial to learned success rates is evaluated for all the participants in both sessions of the experiment. The results (Fig. 4) show a statistically significant increase in the success rate metric across all participants regardless of their movement strategy (effect size = 0.86, *p* < 0.001). Participants learned to perform the kendama task while wearing the exoskeleton, and they were able to do so regardless of the chosen task strategy. There was a statistically significant difference between the initial performance in session A compared to session B (effect size = 0.36, *p* < 0.001), where participants performed better initially in session B. However there was no cross-effect of the session, meaning that the relative change in performance from initial to learned was not affected by the session. This result indicates participants were able to transfer some learning from the first session to the second and were able to then improve upon their performance to the same degree in the second session.

Overall, we find that extrinsic metrics serve as a good measure to compare performance before and after training and that this performance is not hindered by training with an exoskeleton. The improvements in these task-specific metrics for both task domains are indicative of learning. This result is a crucial step in validating the use of the exoskeleton for further in-depth performance analysis.

### Result 2: Exoskeleton wearers converge to certain coordination behaviors during successful attempts

The second milestone in this article is presenting the notion of converging coordination behaviors as a proxy for success and how it is evaluated in different settings. We show that regardless of the task goal, humans converge to joint coordinations that are correlated with success in their post-training or learned attempts. Note that these coordination behaviors may not be consistent between the tasks, or even when using a different strategy within the same task. Yet, participants always demonstrate convergence to their respective success-correlated coordination behaviors.

**Figure 5:**
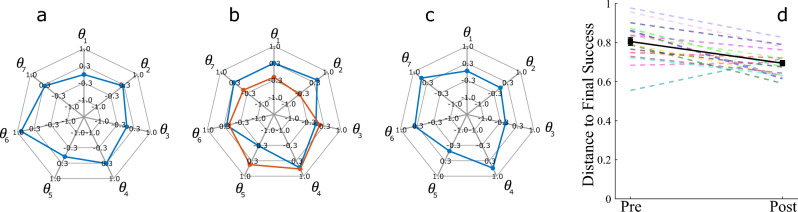
Reach Ninja kinematic coordination distance (KCD) from reference final success. [(**a**) final success, (**b**) and (**c**) non-final successes] are some example coordinations from a reference participant and (**d**) shows the average distance across all participants. Each colored dashed line refers to a different participant. The solid black line with square markers shows the averages across participants in each block and the error bars show the standard error in the pre-training and post-training blocks respectively. On average, participants decrease the KCD from the reference final success from pre-training successes to post-training successes.

**Figure 6:**
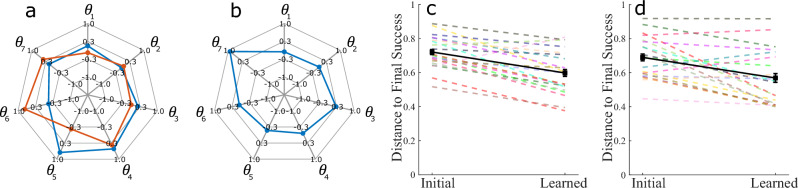
(**a**,**b**) show representative successful coordinations in the final block for two representative participants in their respective session A. (**c**,**d**) show initial and learned distance to reference success for the Virtual Kendama task in session A and session B respectively. Each colored dashed line refers to a different participant. The solid black line with square markers shows the averages across participants in each block and the error bars show the standard error in the initial and learned blocks respectively. In general, participants showed a decreasing trend in distance from reference success from pre/initial performance to post/learned performance in both the Reach Ninja and Kendama tasks.

The improvement in the extrinsic performance of the Reach Ninja and Kendama tasks suggests that participants learn the task and that the behavior in post-training towards the end of the experiment is representative of the learned behavior. Joint angle position data from both studies are used to identify a convergence toward “successful” coordination behaviors. The last successful behavior (representing the most learned behavior) for a given training session and participant is used as a reference coordination and compared to all other successes in the initial and learned attempts in the same session. The kinematic coordination distance metric^35^ (Eq. 1) used to compare two sets of kinematic coordination behaviors from distinct attempts measures how dissimilar the two behaviors are.

1 $$\begin{aligned} D_{P_F,P_X} = min_{i,j} sin(\phi _{i,j}) = min_{i,j} \sqrt{(1 - (u_i \cdot v_i)^2)}, \end{aligned}$$

where $P_F$ and $P_X$ are the principal component coordination behaviors from two different movements F and X with m and n principal components respectively. With $i \in (1,m), j \in (1,n)$. $D_{P_F,P_X}$, this equation gives the minimum distance between any pair of vectors between the two subspaces $P_F$ and $P_X$. A lower distance indicates a higher similarity between the two behaviors. The distance of the final successful coordination $P_F$ (Fig. 5a) from other coordinations $P_A$ and $P_B$ in Fig. 5b,c as measured by Eq. (1) is 0.67 and 0.78 respectively. This metric is evaluated and averaged across each successful movement in the pre-training or initial attempts and the remaining successful movements in the post-training or learned attempts after training. Results for this comparison are demonstrated for the Reach Ninja task in Fig. 5 and the Virtual Kendama task in Fig. 6.

As presented in our prior work^35^, participants in the Reach Ninja study demonstrate a decrease in the kinematic coordination distance from a reference success as learning occurs, decreasing from initial to learned performance ($p < 0.001$). This result indicates that despite the high variability in task performance, participants learn to leverage certain joint coordination behaviors as they improve upon the task. In this article, we additionally consider the Kendama task comparing the final learned successful movement behavior against successes in the initial block and the successes in the final block (except the final success). The coordination distance from the reference behavior also decreases on average from the initial to learned performance (effect size = 0.68, $p < 0.001$). This result is true for both task strategies used by participants, suggesting that they are able to modify their joint coordination for different movement patterns towards the same task goal.

The key contribution in this result is the validation of using kinematic coordination as a measure of performance change in different task domains. Given the differences in the reach ninja and virtual kendama tasks, as well as the different types of virtual kendama strategies, the consistent results demonstrate the robustness of our method for the characterization of kinematic changes in task performance correlated with skill acquisition. Based on the reduction in kinematic coordination distance to the final success in both tasks, we conclude that improvement in extrinsic performance of the human–exoskeleton system is accompanied by an increase in success-correlated joint coordination similarity over time. Specifically, as participants learn a task, regardless of the specific task or movement strategy, they move with increasingly similar joint coordination as they succeed at the tasks. While similar metrics have been used in the past, the goal of this result is to validate the use of extrinsic metrics in the context of training with an exoskeleton. Further, these results provide us with a baseline for the expected change in performance as participants learn. This baseline allows us to identify kinematic measures of performance that are correlated with these extrinsic performance improvements.

**Figure 7:**
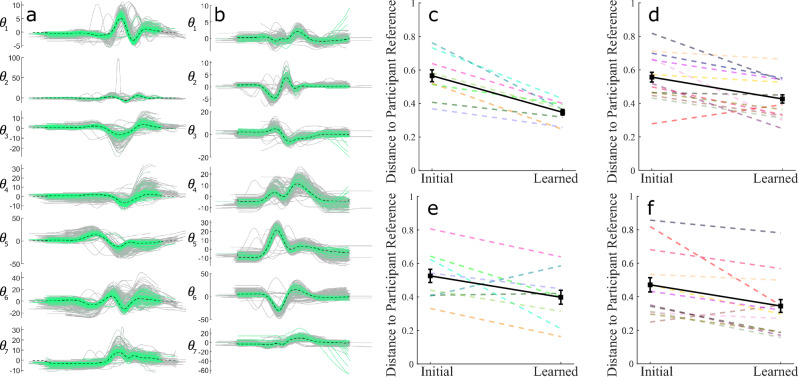
(**a**,**b**) show the end-effector time-correlated displacement in each of the robot’s 7 joint angles in time (zeroed to the start of a movement block for visualization) for a front-swing and a side-wing participant respectively. All gray lines represent unsuccessful attempts whereas green lines represent successful attempts, and the dashed black line represents the average on the final block successes used to construct the reference successful coordination for the two representative participants. The comparison of the constructed reference coordination against initial and non-successful learned coordination are shown in (**c**) front swing session A, (**d**) side swing session A, (**e**) side swing session B, (**f**) front swing session B. In all cases, participants perform more similarly to the reference successful behavior in the learned attempts compared to the initial attempts.

### Result 3: Convergence is a consequence of learning over time

The results from the Reach Ninja and Virtual Kendama kinematic coordination analyses raise an important question about whether the learning process (and not just successes) can be characterized by success-correlated coordination behaviors. Specifically, if participants converge toward certain learned joint coordination behaviors while succeeding over time, is the same behavior observed in all learned block attempts as a result of participants learning the coordination itself? The Reach Ninja task does not allow for the identification of a specific movement attempt when not defined as a success. However, having validated the use of the final success as a reference, and given the more structured Virtual Kendama task, we can compare a reference success-correlated behavior to all attempts of the task regardless of success. To this end, we define a general successful behavior constructed by averaging across all successful movements in the learned block and repeat the analysis performed with only the final successful behavior as a reference. Examples of the averaging process for two representative participants are shown in Fig. 7a,b. By averaging across different successful attempts, we capture more of the variability of the movements and avoid comparing against noisy data from a single attempt. PCA is used on the reference kinematic signals averaged across all successful attempts in the final block, and the resulting kinematic coordination is used as the reference. This reference coordination can be compared against all attempts in a session to identify trends in the data. Note that a similar analysis is impossible to perform with the Reach Ninja task as it is currently designed.

Based on earlier results, we expect that participants will display a decreasing trend in the distance from this generalized reference coordination regardless of whether a given attempt is successful. To capture this trend and confirm statistical significance, we average the distance from the reference coordination across all attempts in the initial block and the failed attempts (all attempts except successes) in the final block. Final block successes are omitted to avoid bias. The averaged distance decreases from initial attempts to learned attempts, as shown in Fig. 7c–f. Repeated measures ANOVA is used to evaluate the change from initial to learned distance (within-subject) from reference coordination across participants in both the *front swing* and *side swing* groups (within-subject) regardless of session number. The results show that there is a statistically significant decrease in the distance (increasing similarity to the reference) from initial to learned behaviors in both groups (effect size = 0.78, *p* < 0.001).

These results show that participants converge to a participant-specific “successful” behavior over time. Specifically, we construct a success-correlated performance using a given participant’s successful movement behaviors and find that the participant does indeed converge to this behavior as they learn. This result only compares the successful behaviors of a given participant against their own performance. However, this method of constructing success-correlated coordination behaviors allows us to further consider the construction of expert successful behaviors as addressed in the next result.

### Result 4: Experts may converge to strategy-specific kinematic coordination

The overarching goal of the current study is to identify avenues for exoskeleton intervention that may benefit the motor learning process. To this end, we aim to identify expert behaviors that can inform the intervention design for novice training. Given the high-dimensional nature of the human–robot system and the dynamic nature of the virtual kendama task, participants could perform a strategy-specific movement using many different coordination behaviors. For example, we observe participants succeed at the side-swing strategy with different coordination behaviors throughout their training, as evidenced by successes that have large KCD relative to their successful coordination (initial performance in Fig. 7). Result 3 suggests that as participants get better at the task, they converge to a specific coordination behavior that may or may not be similar across participants.

**Figure 8:**
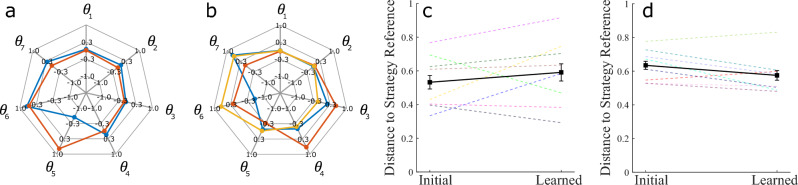
(**a**,**b**) show the strategy-specific coordination behaviors constructed from learned successes (i.e. successful movements in the final 10 attempts of the session) across all participants for the front-swing and side-swing strategies respectively. Kinematic coordination distance (KCD) from strategy-specific coordination is used to compare expert participants against these coordinations as seen in (**c**) for the front-swing and (**d**) for the side-swing. These comparisons omit the attempts that are used to construct the strategy-specific behavior. In the side-swing case, participants perform more similarly to the reference strategy-specific behavior in the learned attempts compared to the initial attempts.

Pilot experiments with the virtual kendama task suggested that participants converged not only to a participant-specific coordination behavior but that this behavior was similar across different participants who became experts at a given strategy. Based on this interesting pilot observation, we hypothesize that we can construct strategy-specific coordination behaviors from a sufficiently large dataset of participants and that expert participants will converge to these behaviors as they learn. We evaluate this hypothesis by first constructing strategy-specific joint coordination behaviors (using data from study 3) and comparing them against the initial and learned block attempts across participants who become experts at the end of training (at least 10 successes in the final block). The successful movements in the last 10 attempts for the first session of every participant are averaged for a given strategy group similar to the averaging process within a participant for the previous result. The strategy-specific averaged joint angle behaviors are then decomposed using PCA. This strategy-specific coordination is then compared to initial and learned performance for experts (at least 10 successes in the learned block). The constructed reference coordinations are represented in Fig. 8a,b, and the results of the comparison are shown in Fig. 8c,d.

Note that these behaviors are not the only way by which success can be achieved, but rather are possible coordination behaviors that we expect expert participants to converge to. An important critique of identifying coordination behaviors across participants is that any common behaviors may be attributed to the participants learning the robot’s dynamics rather than the strategy. However, the distinction between the front-swing and side-swing expert strategy coordination behaviors shows that participants are learning to behave in different ways given strategy-specific instructions. In this condition where participants are instructed to follow a specific strategy, the strategy becomes a part of the task parameters.

We conduct a 2-way repeated measures ANOVA with movement type (front-swing/side-swing) as a between-subject factor and trial type (initial/learned) as a within-subject factor. The analysis does not show a significant effect of the trial type (*p* > 0.05). Note that there are 8 front-swing experts and 8 side-swing experts. There is high variability in the distance to strategy-specific coordinations for the front-swing group suggesting experts were able to succeed with a variety of strategies. However, six expert participants in the side-swing group show a decreasing trend of distance to side-swing coordination from initial to learned performance. To account for high variability, a post-hoc directed paired t-test was conducted for each of the front-swing and side-swing groups. The tests considered initial versus learned performance and specifically queried whether the mean of the Learned data points was lower than that of the Initial data points within a strategy. These t-tests revealed that there was indeed a statistically significant decrease in the distance to strategy reference in the side-swing group (*p* = 0.044) but not in the front-swing group. These results indicate that participants who become experts in the side-swing strategy in their first session tend to converge to the same kinematic coordination over time. More generally, *we identified a strategy specific reference coordination behavior across different participants, and demonstrate that expert participants in one training group tend toward this coordination as they practice the same task goal*.

**Table 1:** p-values for statistically significant comparisons. Note that in this article, p-values are considered significant if they lie below $\alpha = 0.05$. Upon applying Holm-Bonferroni correction the new threshold for significance across 21 hypotheses would be $\alpha = 0.0024$.

Study	Hypothesis	Metric	Factors	p-value
1	H1	Final score percentage	Pre-training Post-training	< 0.001
1	H1	Mean speed	Pre-training Post-training	0.0082
2	H1	Final score percentage	Pre-training Post-training	< 0.001
2	H1	Mean speed	Pre-training Post-training	0.0043
2	H9	KCD to final success	Pre-training Post-training	< 0.001
3	H4	Success rate	Initial Learned	< 0.001
3	H7	Success rate	Session A vs B (initial)	0.01
3	H10	KCD to final success	Initial vs Learned	< 0.001
3	H13	KCD to reference success	Initial vs learned	< 0.001
3	H21	KCD to strategy-specific success	Initial > Learned (Side-swing)	0.009

## Discussion

In this paper, we present evidence that humans learn to perform novel dynamic tasks in an exoskeleton environment by coordinating the robot’s joints in a time-independent but task-dependent manner. Changes in the observed kinematic coordination behaviors and comparisons to reference learned behaviors suggest that participants use feedback in the form of task performance to converge toward success-correlated behaviors over time. Additionally, our results suggest that for certain task goals participants converge to the same coordination behavior as they become experts at the task. Using two task domains, Reach Ninja and Virtual Kendama, and the Harmony exoskeleton, we validate the robustness of our proposed PCA-based kinematic coordination identification method, the kinematic coordination distance metric, and their relevance to the quantification of task-specific expert motor behavior.

We first show that performance metrics such as normalized scores and success rates allow for a superficial assessment of learning^33^. As training progresses, these performance metrics improve, indicating that participants learn to perform the task more accurately and efficiently. We further find that the introduction of the exoskeleton in the learning environment does not significantly hinder learning as evidenced by the null effect of the robot on learned performance. However, we find that these performance metrics provide a partial picture of learning by observing the effect of human behavior on the environment. We complete this picture by characterizing the underlying human behavior using data from the exoskeleton’s sensors. Joint coordination behaviors identified using PCA on the joint angle signals allow for the characterization of intrinsic behavioral changes associated with extrinsic indicators of learning^35^. Thus, we present a comprehensive analysis of both intrinsic and extrinsic performance toward the identification of learning during human–exoskeleton interaction. We also present results that suggest kinematic coordination may be a good representation of locally optimal movement behaviors for a given task goal as observed in expert performers.

In prior work, we presented the use of our intrinsic performance metric for successful movement behaviors for one task domain. In this paper, we validate the prior method in a new task domain demonstrating its robustness, and expand it further to assess general movement behaviors and not just successful ones. Specifically, our results show that the learning trends observed in the reach ninja task are not task-specific or random, as they are also identified in the two sub-cases of the virtual kendama task (side-swing and front-swing). In addition, we present evidence of learning in movements that did not necessarily result in success, thus improving our ability to quantify skill in novice participants. Most importantly, we construct task-specific coordination behaviors that may encode expert domain knowledge per some of our results. Since these coordinations are distinct for the two task instructions (side-swing versus front-swing), we conclude that participants are not only learning the robot dynamics over time but also learning strategy-specific coordination behaviors. We observe that though participants are explicitly instructed to perform a specific strategic movement, we find that success can be achieved for a given strategy with different coordination behaviors. For example, in the front-swing case, not all experts converge to the same across-participant behavior. Thus, the strategy instruction acts as a task parameter, where participants can still perform the task with different coordination behaviors given the same high-level movement goal. However, in the side-swing case, we do find experts converge to the corresponding coordination behavior, suggesting the observed behavior is a preferred solution for that particular strategy. This additional result suggests that for certain task and movement goals, we may quantify across-participant success-correlated coordination behaviors from expert data. The key takeaway from the results of the studies presented in this paper is that as participants learn, they converge toward certain robot joint coordination behaviors correlated with success. Improvements in task performance (score, success/failure) are correlated with convergence to these coordinations. Moreover, certain coordination behaviors may encode ‘﻿optimal’ behaviors for a given task goal as evidenced by similarity across expert participants.

The goal of this article is to systematically validate and test the robustness of kinematic coordination behaviors as a measure of motor behavior and learning in the human–exoskeleton system. To this end, we highlight the need to perform systematic and controlled experiments that validate the robustness of our method. For example, participants in study 3 are given explicit high-level instructions on how to perform the task. The goal of such a controlled study is to establish the usability of kinematic coordination to identify experts for a given strategy. This article provides the necessary validation and serves as a foundation for our future work that will address questions regarding training assessment as well as design.

While the current results present promising evidence in the use of kinematic coordination for skill assessment, they also raise several new questions on the nature of learning and the effect of task parameters. For example, while expert participants converge to similar coordination in the side-swing Virtual Kendama task, the same is not observed in the front-swing Virtual Kendama experts. This discrepancy in results could have two possible explanations. First, the number of expert participants is much lower than the total number of participants since not everyone was able to achieve at least 10 successes in their final training block. Second, there is some indication that participants in the front-swing group (6 experts out of 8 participants) are more successful at the task overall than those in the side-swing group (5 experts out of 13 participants), suggesting that the front-swing strategy is easier and could be performed with more number of distinct coordination behaviors than the side-swing strategy. Due to the scope of the article, we do not focus on answering these kinds of learning-focused questions and instead highlight the possible applications of kinematic coordination in quantifying motor performance. Our future work will look more closely at the observed changes in behavior and analyze these to draw conclusions about the motor learning process.

The convergence of participant behavior to certain measurable task-specific coordinations opens up an interesting new avenue for skill and rehabilitation training design. Prior work suggests that joint coordination is an indicator of motor development and skill^26,36^. Observing and quantifying these coordinations could inform motor training protocol design. Identifying task-specific “expert coordination behaviors” and leveraging them to adaptively modulate robot interaction could potentially bring about persistent behavioral changes in participants^22^. Further, from rehabilitation literature, we know that those suffering from neurological injury benefit from functional motor training^37,38^. Prior work has shown evidence that participants can adapt to environmental constraints introduced by exoskeleton robots, but this adaptation may or may not translate well outside of the exoskeleton after training^1,22,31^. Our current method could be extended to better assess the effect of such training protocols as well as inform the design of novel training protocols. Researchers have also studied the use of coordination behaviors from demonstrations by physical therapists to design exoskeleton interventions for impaired participants^31^. Our work suggests that this method could be taken further to not only provide performance assessment and movement assistance but to identify task-specific “desired coordination behaviors” from expert data and utilize these for the control of exoskeletons for targeted training.

Our overarching goal is to use kinematic coordination as a tool for assessing and designing effective and efficient exoskeleton-based training protocols for physical rehabilitation and skill acquisition. Assuming certain “desirable” kinematic coordination behaviors can be identified for a given task goal by observing experts, and that the exoskeleton interaction can be controlled to teach these coordination behaviors to novices, training protocol design could be made more reactive to the observed behavior. Optimal and adaptive training protocols could then be designed by tailoring the desired movement behaviors to the goal of training (for example, functional task rehabilitation). The same approach could potentially be used to train novices for the efficient use of robotic prostheses or the telemanipulation of surgical robots. Thus, the methods and results presented in this paper lay the foundation for significant advances in human learning-focused robotic interaction control.

## Methods

### Task environments

Two distinct task environments are presented and used for the assessment of human behavior in the exoskeleton. These tasks are designed in particular to satisfy two conditions: (1) to be dynamic and novel to participants; and (2) to provide sufficient challenge for the study of learning.

#### Reach Ninja

Inspired by the popular phone game Fruit Ninja^39^, we present a dynamic reaching game, “Reach Ninja”^33^. The game tracks the movement of an object held in the player’s hand through their webcam (right image in Fig. 2a), and the position is presented on the screen as a circular red marker called the cursor (left image in Fig. 2a). During the game, other circular markers (targets), either blue or black in color, appear on the screen, entering from the bottom edge with randomly chosen initial velocities and radii. The player’s goal is to capture these targets using the red marker, through their hand motion, to maximize their score. The targets are acted upon by a gravitational force, resulting in a predictable projectile motion. The blue targets are assigned a positive score depending on their size (smaller size gives a larger score) and velocity (faster gives a larger score), with the maximum possible score from a positive target being 30. The black targets are assigned a fixed negative score of − 10 independent of size and speed. Two versions of the game are used, a timed version where the task is played for 40 s, and an episodic version with only a fixed number of markers.

To make the task more challenging, we introduce two further dynamic interactions (1) *partial feedback*, where the red cursor is displayed on the screen only briefly, while the hand tracking and game continue normally; and (2) a *virtual magnetic field* around the red marker to repel the positive targets and attract the negative targets. The task difficulty may be modulated using these two interactions. Additionally, we use two versions of this task, a timed one in study 1 (where participants play for 40 s each attempt), and an episodic one in study 2 (where participants only see 3 blue and 2 black markers in a given attempt).

The Reach Ninja task is found to be highly dynamic and supports the study of learning due to its challenging and novel nature. However, the task presents certain additional challenges that make it difficult to capture learning in as much detail as needed for the current research goal. First, the task is inherently variable, making it difficult to compare across different participants and even different attempts by the same participant. Second, the fast, dynamic, and variable movements needed for the task make it impossible to identify attempts that are not characterized as successes. We address these two challenges in a second, virtual reality (VR) based task.

#### Virtual simplified Kendama

The Kendama, a Japanese bowl-and-ball toy, has gained popularity similar to the yo-yo due to its dynamic and challenging nature and the available range of difficulties. The task is simplified to a wide bowl with a ball attached to it through a string (Fig. 2e). The goal of the task is to swing the ball into the cup using the string’s dynamics. The toy satisfies the task requirements to be suitable for our study on the effects of motor training and robot intervention on learned movement behaviors (dynamicity and learnability). A virtual reality version of the Kendama task is designed using Unreal Engine (Epic Games, Cary, NC). The learner dons the Harmony exoskeleton and the Oculus Rift headset (Oculus VR, Menlo Park, CA, Fig. 1a). The Oculus Touch Controller is attached to the end of the robot end-effector and serves as the source of motion for the virtual Kendama.

Like the Reach Ninja task, the Kendama task is inherently dynamic and novel to most participants due to low familiarity with the toy. However, the original Kendama big cup (Oozara) task is fairly challenging to complete with a real Kendama and even harder in the virtual environment. To ensure learnability, we reduce the complexity of the task by replacing the Kendama handle and cup with a large bowl (Fig. 2e). As a result, the task is easier but still sufficiently dynamic so that it is not trivially easy.

Although the Virtual Kendama task is still dynamic and stochastic it offers some advantages over Reach Ninja. Specifically, as there is a single and clear task goal, this task allows comparison across different attempts as well as across different participants. Second, as the task is typically performed in a single swift motion, identification of an attempt is straightforward and allows analysis of movements regardless of whether they were identified as successes.

### Harmony exoskeleton

The Harmony exoskeleton is a bi-manual upper-limb rehabilitation robot^5^ (Fig. 1b). Each arm of the exoskeleton has seven degrees of freedom: (i) shoulder elevation/depression ($\theta _1$), (ii) shoulder protraction/retraction ($\theta _2$), (iii) shoulder abduction/adduction ($\theta _3$), (iv) shoulder internal/external rotation ($\theta _4$), (v) shoulder flexion/extension ($\theta _5$), (vi) elbow flexion/extension ($\theta _6$), and (vii) forearm pronation/supination ($\theta _7$). Torque sensors at each joint are used to control the robot using impedance control^6^. In the gravity assist mode of the robot, also referred to as the transparent mode, the motors compensate for the weight of the robot’s links without compensating for its inertia. The robot passively follows the wearer’s movements, and the resulting environment gives the wearer a sense of mild resistance (similar to moving their arm in water). During run-time, we measure joint angles, joint velocities, and joint torques at each of the seven degrees of freedom. A soft cuff is strapped to the wearer’s upper arm and attached to the robot’s upper arm and the wearer holds the exoskeleton handle. This physical human–robot interaction setup is shown to allow good agreement between the movement of the wearer and the robot end effector and joint angles^6,40^.

### Study designs

Three human-subject studies are presented in this article. The first two are conducted with the Reach Ninja task while the third is conducted with the Virtual Kendama task. The first study is conducted over video conference and does not include the exoskeleton robot. The supplementary video includes clips of participants in all three studies. All experimental protocols are approved by the Institutional Review Board at the University of Texas at Austin. Note that all participants in the following studies are right-handed. Participants provide informed consent prior to the experiment, and all COVID-19 safety guidelines are followed as required by the university. Participants whose images are used to describe the experimental protocols in this paper also gave informed consent for the same.

#### Study 1: Reach Ninja, no robot

10 subjects (6 male, 4 female, aged $27.1 \pm 2.92$) participate in the study^33^. Each participant plays 40 attempts of the timed version of the target Reach Ninja game (Fig. 2a). The target game, including the magnetic field and partial feedback effects, is used to probe the players’ performance at the start, end, and during the experiment. By turning off one of the two interventions, we define two possible source tasks, Partial Feedback Source Task (PFST), and Magnetic Field Source Task (MFST). To study the effect of these source tasks on performance, 5 of the 10 participants (referred to as the curriculum group) are trained on a rudimentary curriculum. The curriculum is ordered as follows: attempts 1–4 are pre-training probe tasks (same as the target task); attempts 5–14 are the PFST (magnetic field off); session 15 is a probe task; attempts 16–25 are MFST (partial feedback off); attempts 26–36 are the target task; and attempts 37–40 are the post-training probe tasks (Fig. 2c). The remaining 5 participants (referred to as the control group) only practice the target task for all 40 attempts. This experiment has been approved by the Institutional Review Board at the University of Texas at Austin under protocol number 2020-07-0156.

#### Study 2: Reach Ninja, with robot

A total of 16 participants (6 female, 10 male, aged 24.4 ± 3.03) are randomly assigned to one of two groups^35^, similar to Study 1. For the ‘targeted practice’ group, training consists of repetitive practice of the target task, whereas for the ‘ordered practice’ group, tasks of varying difficulty levels are practiced with the goal of improving performance on the same target. The targeted and ordered groups are designed to match the control and curriculum groups in study 1 respectively (Fig. 2d). All participants are right-handed and performed the task with their non-dominant (left) arm while wearing the Harmony exoskeleton (Fig. 2b). Prior to its use, the Harmony exoskeleton’s link sizes are adjusted to match robot and body joint locations. At the start of the experiment, each participant dons the Harmony exoskeleton and the tracking object is attached to the end of the exoskeleton handle.

The participant first completes four episodes of a mirrored familiarization task to get accustomed to the robot and game environment. The participant then completes 8 episodes of the target task referred to as the pre-training episodes followed by 308 training episodes. For the ordered practice group, these episodes are ordered as 100 partial feedback source task (PFST) episodes, 4 probe task episodes, 100 magnetic field source task (MFST) episodes, 4 probe task episodes, and 100 target task episodes. Participants in the targeted group practice the same target task for all of the 308 training episodes. The total in-game training duration has been roughly matched to the training duration in study 1 using data from pilot experiments.

Following training, the participant repeats 8 episodes of the target task, referred to as the post-training episodes. To facilitate comparison before and after training, target marker initializations are matched for pre-training and post-training episodes, i.e. the first pre-training is the same as the first post-training episode, and so on. These seeds are randomly selected for each participant before the start of the experiment. This experiment protocol has been approved by the Institutional Review Board at the University of Texas at Austin (STUDY 1215).

#### Study 3: Virtual Kendama, with robot

21 participants (16 male, 5 female aged 21.9 ± 3.6) perform a 2-day human-subject study with the simplified virtual Kendama task (Fig. 2e). Participants are recruited on the basis of low-to-no familiarity ($<=2$ on a Likert scale) with the Kendama task. Each participant attends two separate experimental sessions, each for a duration of 1 h. Prior to its use, the Harmony exoskeleton’s link sizes are adjusted to match robot and body joint locations. Participants are familiarized with the robot-VR environment through random manipulation of a bowl-and-string simulation without the ball attached at the end.

At the start of each session, the participant is asked to employ a specific strategy, either side-swing or front-swing. The order of the strategy for a given participant is randomized. The researcher conducting the experiment ensures the use of the prescribed strategy throughout the experiment. The participant attempts to swing the ball and catch it in the bowl a total 200 times with breaks at increments of 50 attempts (Fig. 2h). At least 1 day’s gap is maintained between two consecutive sessions to allow for the learned behavior to wash out.

### Data analysis

Data is collected from the task as well as the exoskeleton in studies where the exoskeleton is included. For the Reach Ninja task, data is recorded at a variable frame rate depending on how well the host computer is able to run the game. On average the frame rate is at or higher than 15 Hz. For the Kendama task, data is collected from Unreal Engine’s logger at a variable frame rate, usually above 60 Hz. For the Reach Ninja and Harmony experiment, data is collected from the exoskeleton at 20 Hz, whereas for the Kendama task, the collection rate is increased to 1000 Hz. All data is stored in comma-separated text files, and parsed and processed using Matlab (Mathworks, Natick, MA). Piecewise cubic Hermite interpolating polynomial Interpolation is used to increase the signal frequency from 20 to 1000 Hz in all cases.

#### Kinematic coordination from harmony exoskeleton

Principal component analysis (PCA) is a dimensionality reduction method used to analyze multivariate data^41^. Following its success in elucidating kinematic coordinations^22,28,32,42^, this method is chosen to reduce dimensionality in the joint-angle data collected from the Harmony exoskeleton. We identify the principal orthogonal components of the data using singular value decomposition. Next, the percentage of variance in the data that is captured by each principal component is quantified and the components are ordered in decreasing order of variance explained. The first *n* principal components that describe a total majority of the variance (say 90%) are considered to be the primary components and the remaining are discarded. The contributions of each principal component give a sense of linear coordination between the robot’s joints. We refer to these principal components as the principal kinematic coordinations. Further, when these components are identified for successful movements, they are referred to as successful kinematic coordinations.

For the Reach Ninja task, a successful movement is identified as a 0.5s window centered on a positive target capture. For the Kendama task, the start of an attempt for the Kendama task is identified as a velocity peak which is followed by the movement of the ball towards the cup. Success is automatically identified by checking if the distance between the ball and the cup decreases to below a threshold and remains there for at least 1 s.

#### Kinematic coordination distance

Bockemuhl et al.^28^ used PCA to identify joint angle coordination behaviors in catching movements performed by healthy humans. To compare the principal component subspaces from different movements, the authors define a distance metric that measures the amount of rotation required to go from one principal component subspace to the other. However, this metric^28^ is only applicable to comparisons between two subspaces with the same number of principal components. Instead, we require a measure of the *minimum rotation required to align at least one axis of the two subspaces being compared*. Thus, we perform a pairwise comparison of each principal component from two different principal component subspaces (matrices *U* and *V*) using their dot product to get the sine of the angle ($\phi$) between them. This results in a distance matrix of size $m \times n$ where m and n are the number of principal components required to define the first and second subspace respectively. The minimum value in this matrix is calculated as

1 $$\begin{aligned} D_{P_F,P_X} = min_{i,j} sin(\phi _{i,j}) = min_{i,j} \sqrt{(1 - (u_i \cdot v_i)^2}, \end{aligned}$$

where $i \in (1,m), j \in (1,n)$. $D_{P_F,P_X}$, referred to as the principal kinematic coordination distance, represents the minimum distance between any pair of vectors between the two subspaces $P_F$ and $P_X$. As the principal vectors are orthonormal, two different vectors in *U* that are equidistant from *V*, must necessarily be at a distance of $sin(45^{\circ })$ from *V*.

### Statistical analysis

Repeated measures ANOVA is used for statistical evaluation of the results. In each case a p-value below $\alpha = 0.05$ is considered to be significant and allows for the rejection of the null hypothesis in favor of the alternative hypothesis. The specific alternative hypotheses are presented below.

#### Effect of training and exoskeleton on learning of Reach Ninja task

Extrinsic performance measurements for the Reach Ninja task in study 1 and study 2 are used for this analysis. The two factors in the two-way repeated measures ANOVA are attempt type (pre-training versus post-training) within subjects, and training environment (no-robot versus with-robot) between subjects. The following hypotheses refer to either of the two extrinsic metrics, final score percentage and mean speed.

H1 $$\begin{aligned}{} & {} H_{a1}: Pre-training \ne Post-training. \end{aligned}$$

H2 $$\begin{aligned}{} & {} H_{a2}: No-robot \ne With-robot. \end{aligned}$$

H3 $$\begin{aligned}{} & {} H_{a3}: Performance\; Change_{No-robot} \ne Performance\; Change_{With-robot}. \end{aligned}$$

#### Effect of training on learning of Virtual Kendama task

The following hypotheses consider the number of successes in 50 consecutive attempts as the success rate of performing the Virtual Kendama task. The initial block is comprised of the first 50 attempts while the learned block refers to the last 50 attempts. The hypotheses referring to change within a given factor level looks at the change from initial to learned behavior for all data corresponding to that factor level (session number or strategy type).

H4 $$\begin{aligned}{} & {} H_{a4}: Initial \ne Learned.\end{aligned}$$

H5 $$\begin{aligned}{} & {} H_{a5}: Front \ne Side. \end{aligned}$$

H6 $$\begin{aligned}{} & {} H_{a6}: Change_{Front} \ne Change_{Side}. \end{aligned}$$

H7 $$\begin{aligned}{} & {} H_{a7}: SessionA \ne SessionB. \end{aligned}$$

H8 $$\begin{aligned}{} & {} H_{a8}: Change_{SessionA} \ne Change_{SessionB}. \end{aligned}$$

#### Distance from reference successful behavior

Harmony exoskeleton’s kinematic position data from study 2 and study 3 is used in this analysis. The final successful attempt for each participant is considered their reference behavior. This reference behavior is compared to all successful behaviors in pre-training (for Reach Ninja) or initial block (for Virtual Kendama) attempts and all the remaining successes in post-training (Reach Ninja) or learned block (Virtual Kendama) attempts. One-way repeated measures ANOVA is used for study 2, and two-way repeated measures ANOVA is used for study 3. The null hypothesis checks for a change in the kinematic coordination distance from the reference learned behavior from the initial block to the learned block across all participants and sessions.

H9 $$\begin{aligned}{} & {} H_a9: (Pre-training)_{KCD} \ne (Post-training)_{KCD} \end{aligned}$$

H10 $$\begin{aligned}{} & {} H_a10: (Initial)_{KCD} \ne (Learned)_{KCD}\end{aligned}$$

H11 $$\begin{aligned}{} & {} H_a11: (SessionA)_{KCD} \ne (SessionB)_{KCD} \end{aligned}$$

H12 $$\begin{aligned}{} & {} H_{a12}: Change\; in\; SessionA_{KCD} \ne Change\; in\; SessionB_{KCD} \end{aligned}$$

#### Distance from constructed subject-specific reference learned behavior

Harmony exoskeleton’s kinematic position data from study 3 is used for this analysis. All the successful attempts in the learned block of a given session are time-shifted so the peak of the task-space movement in the frontal plane is aligned. The resulting joint-angle signals are averaged in time to generate a reference learned kinematic behavior in the form of a $7\; joint \times T\; timesteps$ matrix. Applying PCA on this reference averaged behavior and extracting the components required to describe at least 90% of the data, we define the reference learned kinematic coordination behaviors for each participant. This learned behavior is compared against all attempts in the initial block and the unsuccessful attempts of the learned block of a given session for each participant. Note that the successes in the learned block are omitted to avoid bias of comparison to the averaged reference behavior. Two independent 2-way repeated measures ANOVA are used for the statistical analysis for each study, where attempt type (pre-training versus post-training) is compared against session type (session A and session B) and strategy type (front-swing versus side-swing). Since all participants performed two sessions of the task, one with each strategy, each of these factors is within-subjects. However, of the 21 participants, 13 performed the side swing strategy on in their first session whereas 8 performed the front swing strategy in their second session. The null hypothesis checks for a change in the kinematic coordination distance from the reference learned behavior from the initial block to the learned block across all participants and sessions.

H13 $$\begin{aligned}{} & {} H_{a13}: Initial_{KCD} \ne Learned_{KCD} \end{aligned}$$

H14 $$\begin{aligned}{} & {} H_{a14}: Front_{KCD} \ne Side_{KCD} \end{aligned}$$

H15 $$\begin{aligned}{} & {} H_{a15}: Change\; in\; Front_{KCD} \ne Change\; in\; Side_{KCD} \end{aligned}$$

H16 $$\begin{aligned}{} & {} H_{a16}: SessionA_{KCD} \ne SessionB_{KCD} \end{aligned}$$

H17 $$\begin{aligned}{} & {} H_{a17}: Change\; in\; SessionA_{KCD} \ne Change\; in\; SessionB_{KCD} \end{aligned}$$

#### Distance from constructed strategy-specific reference learned behavior

Harmony exoskeleton’s kinematic position data from study 3 is used for this analysis. All the successful movements in the last 10 attempts of the first session are time-shifted so the peak of the task-space movement in the frontal plane is aligned. These joint-angle signals are averaged across all participants using the same strategy to generate a reference strategy-specific kinematic behavior in the form of a $7\; joint \times T\; timesteps$ matrix. We define the reference strategy-specific coordination as the first principal component of this averaged startegy-specific behavior determined using PCA. This strategy-specific behavior is compared against all attempts in the initial block and the attempts of the learned block not included in the construction of the reference behavior for expert participants. Note that expert participants are defined as those who achieve at least 10 successes in their learned block (20% success rate). A 2-way repeated measures ANOVA is used for the statistical analysis where attempt type (initial versus learned) is a within-subject factor and strategy type (front-swing versus side-swing) is a between-subject factor. We identify 5 side-swing experts and 6 front-swing experts. The null hypothesis checks for a change in the kinematic coordination distance from the reference learned behavior from the initial block to the learned block across all expert participants in their respective first sessions.

H18 $$\begin{aligned}{} & {} H_{a18}: Initial_{KCD} \ne Learned_{KCD} \end{aligned}$$

H19 $$\begin{aligned}{} & {} H_{a19}: Front_{KCD} \ne Side_{KCD} \end{aligned}$$

H20 $$\begin{aligned}{} & {} H_{a20}: Change\; in\; Front_{KCD} \ne Change\; in\; Side_{KCD} \end{aligned}$$

For post-hoc analysis, we also use a directional paired t-test within a strategy. Specifically, this test checks whether the mean of the initial group distance is higher than the mean of the learned group distance to strategy-specific coordinations.

H21 $$\begin{aligned}{} & {} H_{a21}: Initial_{KCD} > Learned_{KCD} \end{aligned}$$

## Supplementary Information

Supplementary Information 1.

Supplementary Information 2.

## Data Availability

The datasets used and/or analyzed during the current study are available from the corresponding author on reasonable request.
