# Supplementary material for: Kinematic coordinations capture learning during human–exoskeleton interaction
Source: Sci Rep. 2023 Jun 26;13:10322. doi: 10.1038/s41598-023-35231-3 (PMC10293206; doi:10.1038/s41598-023-35231-3)
Supplement: Supplementary file 2 — Supplementary Information 2. [file 41598_2023_35231_MOESM2_ESM.docx]

Demonstration of experimental conditions. The three videos show participants performing each of the three experiments. The first video shows the reach ninja no robot (RNNR) case, the second shows the reach ninja with robot (RNWR) case and the third shows the virtual kendama with robot (VKWR) case.
